# Breast cancer risks in women with a family history of breast or ovarian cancer who have tested negative for a BRCA1 or BRCA2 mutation

**DOI:** 10.1038/sj.bjc.6604830

**Published:** 2008-12-16

**Authors:** K A Metcalfe, A Finch, A Poll, D Horsman, C Kim-Sing, J Scott, R Royer, P Sun, S A Narod

**Affiliations:** 1 Lawrence S. Bloomberg Faculty of Nursing, University of Toronto, Toronto, ON, Canada; 2 Women's College Research Institute, Toronto, ON, Canada; 3 BC Cancer Agency, Vancouver, BC, Canada

**Keywords:** BRCA1, BRCA2, uninformative results, cancer risks

## Abstract

Genetic testing for mutations in BRCA1 and BRCA2 is available in Canada for women with a significant family history of breast cancer. For the majority of tested women, a BRCA1 or BRCA2 mutation is not found, and counselling regarding breast cancer risk is based on the review of the pedigree. In this prospective study, we estimate breast cancer risks in women with a family history of breast cancer and for whom the proband tested negative for a mutation in BRCA1 or BRCA2. Families with two or more breast cancers under the age of 50 years, or with three cases of breast cancer at any age, and who tested negative for a BRCA1 or BRCA2 mutation were identified. Follow-up information on cancer status was collected on all first-degree relatives of breast cancer cases. The standardised incidence ratios (SIRs) for breast cancer were calculated by dividing the observed numbers of breast cancer by the expected numbers of breast cancers, based on the rates in the provincial cancer registries. A total of 1492 women from 365 families were included in the analyses. The 1492 first-degree relatives of breast cancer cases contributed 9109 person-years of follow-up. Sixty-five women developed breast cancer, compared to 15.2 expected number (SIR=4.3). The SIR was highest for women under the age of 40 (SIR=14.9) years and decreased with increasing age. However, the absolute risk was higher for women between the age of 50 and 70 (1% per year) years than for women between 30 and 50 (0.4% per year) years of age. There was no elevated risk for ovarian, colon or any other form of cancer. Women with a significant family history of breast cancer (ie, two or more breast cancers under the age of 50 years, or three or more breast cancers at any age), but who test negative for BRCA mutations have approximately a four-fold risk of breast cancer. Women in these families may be candidates for tamoxifen chemoprevention and/or intensified breast screening with an MRI.

A woman's breast cancer risk is increased if she has a first-degree relative with breast cancer at a young age, or if she has more than one relative with breast cancer (Claus *et al*, 1990; Cancer, Collaborative Group on Hormonal Factors in Breast Cancer (CGoHFiB), 2001). The Collaborative Group on Hormonal Factors in Breast Cancer (Cancer, Collaborative Group on Hormonal Factors in Breast Cancer (CGoHFiB), 2001) analysed data from 52 epidemiological studies and reported on the risks of breast cancer in the first-degree relatives. The estimated cumulative incidence of breast cancer up to the age of 80 years for individuals with zero, one, and two first-degree relatives were 7.8, 13.3, and 21.1%, respectively (Cancer, Collaborative Group on Hormonal Factors in Breast Cancer (CGoHFiB), 2001).

For women with a significant family history of breast cancer, genetic testing for mutations in BRCA1 and BRCA2 is available throughout Canada and elsewhere. Approximately, 25% of familial breast cancer patients carry a mutation in one of the two breast cancer genes (Shih *et al*, 2002; Simard *et al*, 2007). Hence, the majority of women with a family history of breast cancer will receive a negative genetic test result. For these women, counselling regarding breast cancer risk estimation is difficult and is based on the examination of the pedigree and the evaluation of other risk factors. It is unclear to what extent their breast cancer risk is raised. In this study, we estimate the breast cancer risks for women with a strong family history of breast cancer, but tested negative for a mutation in BRCA1 or BRCA2.

## Materials and methods

### Sample

Eligible subjects were drawn from a database of families who had received testing for BRCA1 and BRCA2 mutations in Ontario and British Columbia, but tested negative. Each family contained two or more breast cancers diagnosed under the age of 50 years, or three cases of breast cancer diagnosed at any age. These families were assessed for genetic risk at one of the two Canadian centres between 1993 and 2003. In each family, at least one woman affected with breast cancer had been tested and was found not to carry a BRCA1 or BRCA2 mutation. Testing methods varied between the centres and over time; but in each case, all coding exons of BRCA1 and BRCA2 were screened using a combination of PTT, DGGE, dHPLC, and direct sequencing.

### Methods

Eligible individuals within these families included all female first-degree relatives of the breast cancer cases who were over the age of 18 years at the time when the pedigree was drawn. Each pedigree was dated on the day in which the information was collected. The proband (first person from family to make contact with genetics centre) from each family was contacted by telephone. The family history was updated through a telephone interview. Information was collected on all female first-degree relatives of all breast cancer cases within the family. Collected data included age, cancer status, prophylactic surgery, and vital status. In the event that the proband was not familiar with the family history in sufficient detail, we asked her to identify a second person in the family who was able to provide additional details. The study received ethics approval from both participating centres.

### Analysis

All first-degree relatives of women affected with breast cancer in the family were identified. These women were considered as a cohort of at-risk women, and the risk of breast and other cancers were estimated in the cohort using survival analysis. Each woman was considered to be at risk of cancer from the date of the original pedigree ascertainment to the earliest of (1) the date of the follow-up telephone call; (2) the date at which cancer was diagnosed (if diagnosed with cancer), or (3) the date of death (if deceased). For each study subject, the total number of person-years at risk was calculated. Person-years at risk were then categorised according to age group, in 5-year age groupings. The annual cancer risk was estimated for the entire cohort, for each of the 5-year groupings, by dividing the number of cancers observed by the total number of person-years at risk, and multiplying by 10^5^. The expected rates for Ontario and for British Columbia were obtained from the registry data recorded in *Cancer Incidence in Five Continents* (volume VII). The SIR was estimated from the ratio of the observed rate to the expected rate. We estimated the cumulative incidence of cancer in the cohort of first-degree relatives by applying the observed age-specific rates to a theoretical cohort of women from 25 to 75 years of age.

## Results

Among the 474 families, there was a total of 874 breast cancers (mean: 51.0, range: 25–90) were identified in the pedigrees at baseline. We identified 1599 first-degree relatives of the 874 women with breast cancer and who did not have breast cancer at the time of the initial ascertainment. Data were missing for 423 women. For 107 women, the proband who has tested was unaffected and they were excluded. The remaining 1492 were members of a family in which an affected woman (i.e., with a previous diagnosis of cancer) had undergone testing for BRCA1 and BRCA2. The analysis is restricted to these 1492 women from 365 families. The mean age of these women at the time of pedigree drawing was 48.2 years (range: 17–99) and at the time of the follow-up interview was 54.3 years (range: 24–101). The mean time from genetic testing in the proband to the time of follow-up was 6.1 years (range: 1–10). The 1492 first-degree relatives contributed 9109 person-years of follow-up.

**Figure 1:**
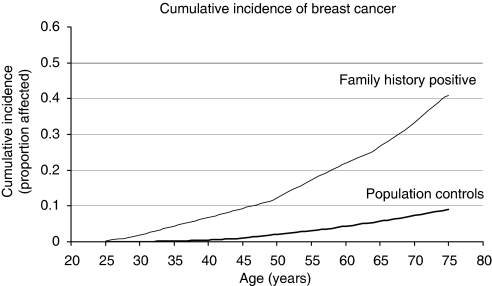
Cumulative incidence of breast cancer.

A total of 65 women developed breast cancer, compared to an expected number of 15.2. The actuarial risk of breast cancer in the entire cohort was 0.7% per year, or roughly 40% over the lifetime (Figure 1). The annual risk increased from 0.4% per year for women between 25 and 40 years of age to approximately 1% per year for women over the age of 50 years (Table 1). The expected number of breast cancers were determined from age-specific cancer incidence rates recorded in *Cancer Incidence in Five Continents* (Vol. VIII, IARC). The standardised incidence ratios (SIRs) for breast cancer were calculated by dividing the observed numbers of breast cancer by the expected numbers of breast cancer. Table 1 presents the SIRs by age at time of baseline (i.e., genetic testing in proband). The SIRs decreased with increasing age (Table 2), ranging from 14.9 at age of 25–39 years to 3.0 at age 60 years or older.

Subanalyses were conducted based on the number of breast cancers in relatives (Table 3). The observed number of breast cancers was significantly greater than expected for all subgroups. There was no increase in SIR with increasing number of relatives with breast cancer. A separate analysis was conducted on the basis of the number of first-degree relatives with breast cancer diagnosed at age of 50 years or younger (Table 4). Again, there were no differences according to the number of relatives with early-onset breast cancer. The SIRs were also calculated for other cancers (Table 5). There was no significant difference in observed *vs* expected number of ovarian cancers (*P*=0.82) or colon cancers (*P*=0.79). The excess cancer risk was limited to breast cancer.

## Discussion

In Ontario and British Columbia, women with a strong family history of breast cancer are eligible for genetic testing for BRCA1 and BRCA2. However, for most families who exhibit the characteristics of hereditary transmission of breast cancer, a BRCA1 or BRCA2 mutation is not detected. For women in these families, questions remain about their breast cancer risk and the appropriate risk management strategies. In this study, we report that women with a family history of breast cancer (i.e., two or more breast cancers under the age of 50 years, or three or more breast cancers at any age) are at elevated risk of breast cancer. In many centres, a negative BRCA report is reported as ‘non-informative’ (i.e., no mutation can be detected). This implies that the risk of cancer is most likely higher than those with a ‘true negative’ (i.e., those with a negative genetic test result in a family with a known BRCA1 or BRCA2 mutation) genetic test result. However, it is unclear to what extent a woman's cancer risk is increased when faced with a ‘non-informative’ genetic test result. Our study confirms that women with a family history of breast cancer do have an increased risk of breast cancer; however, it should be noted that there was no evidence for an increase in the risk of ovarian or any other cancer. Surveillance and preventive measures should be directed towards breast cancer.

In an earlier study, Kauff *et al* (2005) reported on breast and ovarian cancer risk in 165 BRCA1 and BRCA2 mutation-negative hereditary breast cancer families (Kauff *et al*, 2005). They reported an SIR for breast cancer that was similar to ours (SIR=3.1, 95% CI=1.9–4.9; *P*<0.001). Again, no significantly increased risk was seen for ovarian cancer (SIR=1.5, 95% CI=0.02–8.5; *P*=0.5). However, age-specific risks were not presented (likely due to the small sample size). In addition, 23% of the probands in their study were unaffected with breast cancer. This may explain why the SIR for breast cancer reported by Kauff *et al*. (2005) was lower than ours (SIR=4.3).

In our study, women younger than the age of 40 years had the greatest elevation in breast cancer risk, compared to age-specific general population breast cancer risks. After the age of 40 years, the breast cancer relative risks were less extreme. Olsen *et al* (1999) reported SIRs for breast cancer in mothers and sisters of 2840 probands with early-onset breast cancer (under the age of 40 years; mothers: SIR=2.0; 95% CI=1.8–2.2; sisters: SIR=2.6; 95% CI=2.1–3.2; Olsen *et al*, 1999). The SIR was influenced by the age of the female relative, with younger women having higher risks of breast cancer. Rawal *et al* (2006) reported similar findings among 31 235 first-degree relatives of 8807 probands with early-onset breast cancer (SIR=2.3: 95% CI=2.0–2.7). (Rawal *et al*, 2006). In the largest study, the Cancer, Collaborative Group on Hormonal Factors in Breast Cancer (CGoHFiB) (2001) estimated the relative risk for women with three or more first-degree relatives with breast cancer to be 3.9 (95% CI=2.0–7.5). This estimate is very similar to that for the women in our cohort (SIR=4.3). However, we estimate the lifetime risk for breast cancer to be 40% compared to 21% in the Collaborative Group study.However, in these studies, BRCA1 and BRCA2 testing were not conducted and mutation carriers were not excluded.

Recently, there has been much interest in generating and applying polygenic risk models for risk assesment in breast cancer; these are based on developing a profile of genotypes for a number of single nucleotide polymorphisms. For example, Pharoah *et al* (2008) propose that a seven-gene profile can help explain a proportion of the heritability of breast cancer in the population and can be used to assign women to different levels of risk. The approach has been criticised by others as having a limited ability to discriminate risk in women in the general population (Gail, 2008). The odds ratios associated with a ‘high-risk’ profile in the polygenic model tend to be approximately 1.5 compared to the odds ratio of 4 that we observed, based on the family history alone. In our opinion, family history is a much more useful tool than are polygenic tests and more effort should be concentrated on refining risk estimates by conducting more studies such as ours. Greater attention should be paid by the medical community in order that a detailed and accurate family history of breast cancer is obtained on all women.

For women with a BRCA1 or BRCA2 mutation, current options include breast screening, prophylactic surgery, and chemoprevention. However, in women from high-risk families in which a BRCA1 or BRCA2 mutation is not detected, it is unclear if the screening and risk reduction options offered to these women should be the same as for women with a BRCA1 or BRCA2 mutation. MRI has been shown to be effective in detecting small breast cancers in BRCA1 and BRCA2 mutation carriers (Warner *et al*, 2004; Leach *et al*, 2005; Sardanelli *et al*, 2007). The American Cancer Society recommends an annual MRI screening to women with a BRCA mutation, to women with a first-degree relative with a BRCA mutation (but untested), and to women with a lifetime risk of 20–25% or greater. Therefore, on the basis of this study, women in a hereditary breast cancer family without a BRCA mutation may qualify for MRI screening based on their lifetime risk of breast cancer. We estimate the lifetime risk of breast cancer in these women to be approximately 40%, or roughly one-half of a BRCA1 or BRCA2 mutation carrier. Therefore, when counselling women from families, such as these, breast screening using MRI should be considered.

Tamoxifen is another option. In the National Surgical Adjuvant Breast and Bowel Project P-1 Study, tamoxifen reduced the risk of invasive breast cancer by 49% in women at high risk (Fisher *et al*, 1998). High risk was defined as having a breast cancer risk of greater than 1.66% in the next 5 years. All of the women in this study met this criterion; from 40 to 45 years of age, the risk for cancer was 2.5% and from 50 to 55 years of age it was approximately 5%. Therefore, tamoxifen should be presented as an option for women in these high-risk families without a BRCA mutation. It would be of interest to know the estrogen-receptor status of the newly diagnosed breast cancers in our study to better estimate the potential for tamoxifen to decrease breast cancer risk.

The principal strength of our study is that this is a prospective cohort and the risk estimates were based on cancers diagnosed after the family was ascertained. This strategy eliminates the possibility of ascertainment bias, which is often a concern when risk estimates are based on family histories obtained at the time the family is identified. A second strength of our study is that we have excluded families with a known BRCA1 or BRCA2 mutation, and therefore our risk estimates should be valid for women who are referred for genetic testing and who receive a negative test result. There are limitations to our study. All cancers that were documented were self-reported and were not confirmed by pathology report. The genetic testing was limited to the proband and the incident cases of cancer in the study did not have genetic testing. It is possible that, in some cases, the proband was a sporadic case in a family where a mutation was present. It is also possible that we missed a small number of BRCA mutations. However, given that we saw no excess risk of ovarian cancer in the study, it is unlikely that this is an important factor. We excluded 423 of 1599 potential study subjects (26%), because the proband did not have information on their clinical and/or vital status. In general, these were more distant relatives. However, if the proband selectively included relatives with breast cancer, then this selection bias might result in a spuriously elevated SIR.

In conclusion, women from high-risk breast cancer families in which a BRCA1 or BRCA2 mutation can not be found need to be counselled about their increased risk for breast cancer. Although this risk is not as high as that for BRCA1 and BRCA2 mutation carriers, it is significantly higher than the general population risk and appropriate breast screening, and prevention should be recommended.

## Figures and Tables

**Table 1:** Standardised incidence ratios (SIRs) of breast cancer by age

		**Age at baseline**												
**Variables**	**Total**	**25−**	**30−**	**35−**	**40−**	**45−**	**50−**	**55−**	**60−**	**65−**	**70−**	**75−**	**80−**	**85+**
Frequency	1492	171	152	176	214	167	144	108	86	63	76	56	44	35
Person-years	9109	738	794	1032	1215	1183	1003	737	579	467	438	364	291	268
New breast cancers Observed (*N*)	65	2	4	5	6	6	10	7	5	6	7	4	1	2
New breast cancers expected (*N*)	16.49	0.05	0.16	0.53	1.23	2.06	2.17	1.85	1.64	1.60	1.61	1.45	1.15	0.99
Rate per 10^5^ observed	713.6	271.0	503.8	484.5	493.8	507.2	997.0	949.8	863.6	1284.8	1598.2	1098.9	343.6	746.3
Rate per 10^5^ expected	181.0	6.8	20.8	51.5	101.4	173.9	216.6	250.8	284	342.7	366.9	397.6	393.7	369.1
SIR	3.9	40.1	30.3	9.4	5	2.9	4.1	3.8	3.1	3.8	5	2.8	0.9	2.1
95% CI	3.1–5.0	11.0–146	7.8–51	4.3–23.4	2.3–10.9	1.3–6.2	2.5–8.4	1.9–8.0	1.3–7.3	1.7–8.2	2.1–9.0	1.1–7.3	0.2–5.1	0.6–7.3
*P-*value	<0.0001	0.02	0.002	0.002	0.007	0.05	0.0008	0.009	0.05	0.02	0.006	0.10	0.92	0.41

**Table 2:** Standardised incidence ratios (SIRs) of breast cancer by age group

**Age group**	**Observed (rate per 10^5^)**	**Expected (rate per 10^5^)**	**SIRs**	**95% CI**	***P*-value**
25–39	11 (42.9)	0.74 (2.9)	14.9	8.30–26.6	<0.0001
40–49	12 (50.0)	3.29 (13.7)	3.64	2.09–6.38	0.001
50–59	17 (97.7)	4.02 (23.1)	4.23	2.64–6.77	<0.0001
60 and above	25 (103.9)	8.44 (35.1)	2.96	2.01–4.37	<0.0001

**Table 3:** Standardised incidence ratios (SIRs) of breast cancer by the number of relatives diagnosed with breast cancer at any age

**Number of relatives diagnosed with breast cancer**	**Mean age at baseline (range)**	**Observed (rate per 10^5^)**	**Expected (rate per 10^5^)**	**SIRs**	**95% CI**	***P*-value**
1 or 2 (*n*=182)	45.9 (17–85)	9 (790.9)	1.88 (165.2)	4.79	2.52–9.10	0.001
3 (*N*=479)	49.6 (18–92)	23 (779.1)	5.57 (188.7)	4.13	2.75–6.20	<0.0001
4 or more (*N*=830)	47.9 (18–99)	32 (638.3)	9.02 (179.9)	3.55	2.51–5.01	<0.0001

**Table 4:** Standardised incidence ratios (SIRs) of breast cancer by the number of first-degree relatives diagnosed with breast cancer of ⩽50 years

	**Observed (rate per 10^5^)**	**Expected (rate per 10^5^)**	**SIRs**	**95% CI**	***P*-value**
No first-degree relative with breast cancer of ⩽50 years (*N*=607)	25 (678.2)	7.11 (192.9)	3.52	2.38–5.19	<0.0001
One first-degree relative with breast cancer of ⩽50 years (*N*=677)	27 (646.9)	6.29 (150.7)	4.29	2.95–6.25	<0.0001
Two or more first-degree relatives with breast cancer of ⩽50 years (*N*=207)	12 (965.4)	3.08 (247.8)	3.90	2.23–6.81	0.0006

**Table 5:** Standardised incidence ratios (SIRs) of other cancers

**Cancer type**	**Observed (rate per 10^5^)**	**Expected (rate per 10^5^)**	**SIRs**	**95% CI**	***P*-value**
Breast cancer	65 (713.6)	16.49 (181.0)	3.94	3.09–5.02	<0.0001
Ovarian cancer	2 (21.5)	2.34 (23.3)	0.85	0.23–3.12	0.82
Colon cancer	4 (43.1)	5.04 (48.9)	0.79	0.31–2.04	0.79
All cancers	98 (1083.7)	54.14 (552.5)	1.81	1.49–2.21	<0.0001
All cancers except breast cancer	33 (364.9)	37.78 (385.8)	0.87	0.62–1.23	0.42

Skin cancer is not included as a cancer.
